# Environmental responsiveness of tubulin glutamylation in sensory cilia is regulated by the p38 MAPK pathway

**DOI:** 10.1038/s41598-018-26694-w

**Published:** 2018-05-30

**Authors:** Yoshishige Kimura, Koji Tsutsumi, Alu Konno, Koji Ikegami, Saira Hameed, Tomomi Kaneko, Oktay Ismail Kaplan, Takayuki Teramoto, Manabi Fujiwara, Takeshi Ishihara, Oliver E. Blacque, Mitsutoshi Setou

**Affiliations:** 1 Department of Cellular & Molecular Anatomy, Hamamatsu University School of Medicine, 1-20-1 Handayama, Hamamatsu Shizuoka, 431-3192 Japan; 2 Department of Liberal Arts and Sciences, Kanagawa University of Human Services, 1-10-1 Heisei-cho, Yokosuka, Kanagawa 238-8522 Japan; 3 Department of Anatomy and Developmental Biology, Graduate School of Biomedical and Health Sciences, Hiroshima University, 1-2-3 Kasumi, Minami-ku, Hiroshima, Hiroshima, 734-8553 Japan; 4 School of Biomolecular and Biomedical Science, University College Dublin, Belfield, Dublin, 4 Ireland; 5 Department of Biology, Faculty of Sciences, Kyushu University, 744 Motooka, Nishi-ku Fukuoka, 819-0395 Japan; 6 Department of Systems Molecular Anatomy, Institute for Medical Photonics Research, Preeminent Medical Photonics Education & Research Center, Hamamatsu University School of Medicine, 1-20-1 Handayama, Hamamatsu Shizuoka, 431-3192 Japan; 7 The Institute of Medical Science, The University of Tokyo, 4-6-1 Shirokanedai, Minato-ku, Tokyo 108-8639 Japan; 8 Department of Anatomy, The University of Hong Kong, Hong Kong, China; 9 Division of Neural Systematics, National Institute for Physiological Sciences, 38 Nishigonaka Myodaiji, Okazaki, Aichi 444-8585 Japan; 10 Present Address: Division of Cell Biology, Department of Biosciences, School of Science, Kitasato University, 1-15-1 Kitasato, Minami-ku, Sagamihara, Kanagawa 252-0373 Japan; 11 Present Address: Abdullah Gul Universitesi, Doga Bilimleri Fakultesi, Sumer Kampusu, 38090 Kocasinan Kayseri, Turkey

## Abstract

Glutamylation is a post-translational modification found on tubulin that can alter the interaction between microtubules (MTs) and associated proteins. The molecular mechanisms regulating tubulin glutamylation in response to the environment are not well understood. Here, we show that in the sensory cilia of *Caenorhabditis elegans*, tubulin glutamylation is upregulated in response to various signals such as temperature, osmolality, and dietary conditions. Similarly, tubulin glutamylation is modified in mammalian photoreceptor cells following light adaptation. A tubulin glutamate ligase gene *ttll-4*, which is essential for tubulin glutamylation of axonemal MTs in sensory cilia, is activated by p38 MAPK. Amino acid substitution of TTLL-4 has revealed that a Thr residue (a putative MAPK-phosphorylation site) is required for enhancement of tubulin glutamylation. Intraflagellar transport (IFT), a bidirectional trafficking system specifically observed along axonemal MTs, is required for the formation, maintenance, and function of sensory cilia. Measurement of the velocity of IFT particles revealed that starvation accelerates IFT, which was also dependent on the Thr residue of TTLL-4. Similarly, starvation-induced attenuation of avoidance behaviour from high osmolality conditions was also dependent on *ttll-4*. Our data suggest that a novel evolutionarily conserved regulatory system exists for tubulin glutamylation in sensory cilia in response to the environment.

## Introduction

Glutamylation, a post-translational modification (PTM) found on tubulin and other substrates^1,2^, is performed by a reversible regulatory mechanism controlled by evolutionarily conserved enzymatic systems. Members of the tubulin tyrosine ligase-like (TTLL) family^2–4^ function as tubulin glutamate ligases, which add multiple glutamate moieties to specific glutamate residues within substrate proteins. Conversely, side chain glutamates are removed by tubulin deglutamylases of the cytoplasmic carboxypeptidase (CCP) family^5,6^.

**Figure 1:**
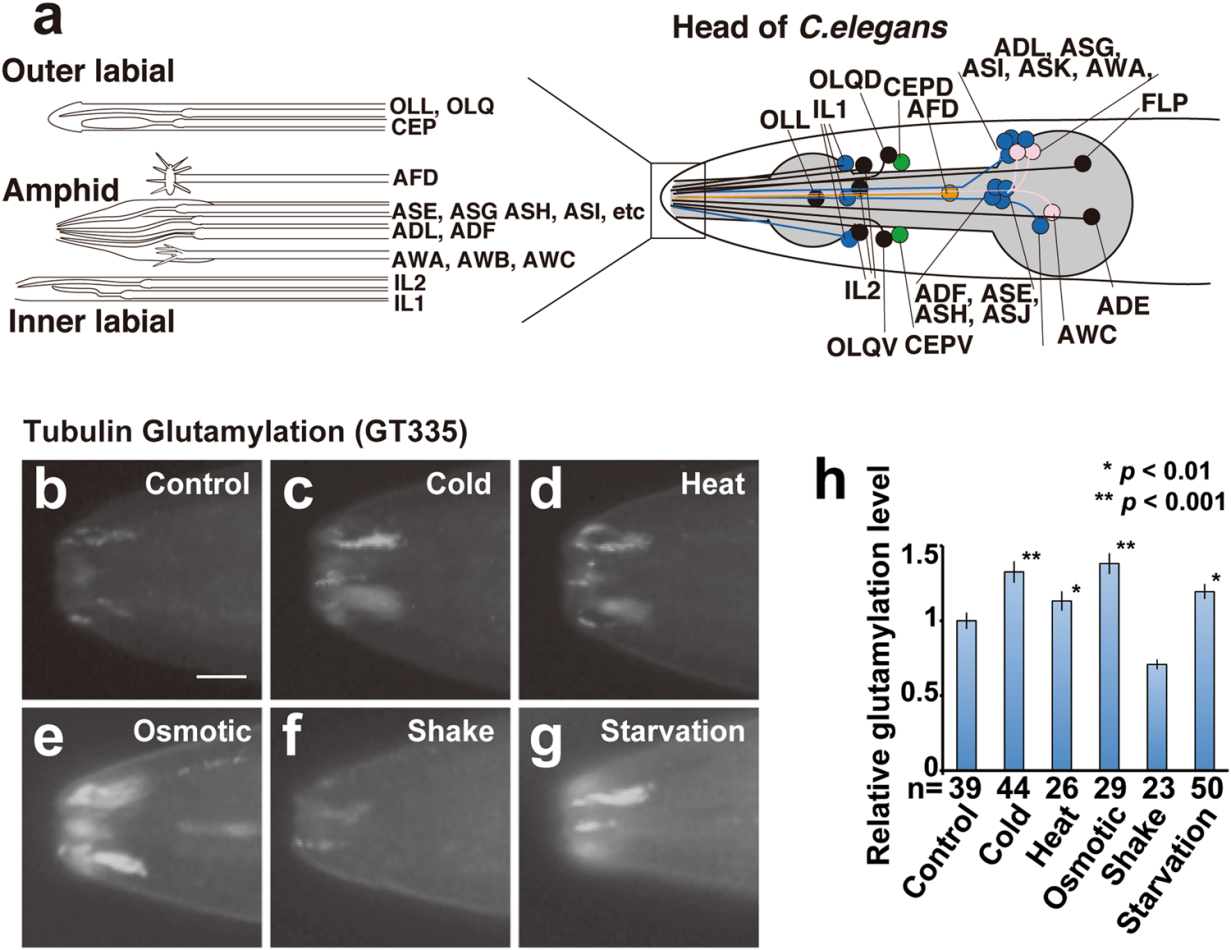
Tubulin glutamylation in *C. elegans* sensory cilia is modulated by the environment. (**a**) Schematic diagram of a worm head that illustrates the positions of the ciliated cell bodies (right) and their ending (left). (**b**–**g**) Results of immunohistochemical analysis of the *C. elegans* head (around tip of nose) using GT335, which recognises glutamylated tubulin. Signals can be observed in the cilia of amphid, inner labial, and outer labial neurons. Signal intensities were compared between control (**b**) and worms exposed to environmental stimuli (**c**–**g**). (**h**) Quantified result of tubulin glutamylation described in (**b**–**g**). All stimuli except physical vibration induced a significant increase in tubulin glutamylation. Asterisks (*p < 0.01, Student’s t test) and double asterisks (**p < 0.001, Student’s t test) indicate significant differences as compared with the wild-type. Environmental stimuli of “heat”, “cold”, “osmotic”, and “starvation” but not of “shake” induced a significant increase in glutamylation. Numbers of animals scored are indicated (h, bottom). Bars indicate mean ± S.E. Scale bars = 5 μm.

We employed the genetically tractable roundworm, *Caenorhabditis elegans*. Specifically, we investigated tubulin PTM in *C. elegans* sensory cilia, which are microtubule (MT)-based organelles that extend from the distal dendrite tips of 60 sensory neurons found in sensory organs named amphid (head) and phasmid (tail) sensilla (Fig. 1a).

In *C. elegans*, tubulin glutamylation is predominantly observed in axonemal MTs of non-motile cilia located at the ends of dendritic processes of sensory neurons. We and another group have reported the molecular identity of ciliary tubulin glutamylase and deglutamylase enzymes in *C. elegans*^5–7^. There are six *ttll* genes (*ttll-4*, *-5*, *-9*, *-11*, *-12*, and *-15*) and two *ccp* genes (*ccpp-1*, and *-6*) in the worm genome. Loss of *ccpp-6* results in elevated levels of ciliary MT glutamylation. Consistent with a deglutamylase function for CCPP-6, overexpression of this protein in ciliated cells decreases glutamylation signals^5^. Using murine CCP5, orthologous to CCPP-6 in worms, we found that recombinantly expressed CCP5 exhibits deglutamylase activities *in vitro*^5^. In zebrafish, knockdown of an orthologue of CCP5 also increases ciliary tubulin glutamylation and induces ciliopathy phenotypes^8^. It is thought that the diversity of MT subtypes generated by tubulin glutamylation is maintained by balanced activities of TTLL glutamylases and CCP deglutamylases. However, the physiological plasticity and regulatory mechanisms underpinning tubulin glutamylation are not well understood.

Cilia are evolutionarily conserved MT-based organelles extending from the surface of most eukaryotic cells. Classified as motile or non-motile (primary cilia), these organelles serve critical cellular functions in cell and fluid motility, as well as sensory and developmental signal transduction. Cilium formation and maintenance is dependent on intraflagellar transport (IFT), which operates bidirectionally along the ciliary MTs, driven by anterograde (ciliary base to tip) kinesin-2 and retrograde (ciliary tip to base) cytoplasmic dynein motors. IFT is a primary mechanism by which structural (e.g. tubulin) and functional (e.g. signalling molecules) ciliary subunits are transported to and distributed within cilia^9^.

There are several reports that describe the relationship between tubulin modification and protein/vesicular transport along neuronal MTs. In mammalian neurons, the activities of a number of kinesin motors are known to be regulated by modified forms of MTs^10^. For example, tyrosinated tubulin, which is enriched in dendrites, alters the interaction between kinesin-1 and MTs, causing kinesin-1 to navigate into axonal processes^11^. In addition, tubulin glutamylation regulates KIF1A entry into neurites, enabling it to effectively transport cargo to target locations^10^. In *C. elegans*, the CCPP-1 deglutamylase is required for the stability of a subset of sensory cilia, and also regulates the ciliary localisation of kinesin-3 KLP-6 and the speed of the kinesin-2 KIF17 homologue OSM-3^7^.

The molecular mechanisms by which external environmental signals regulate tubulin PTMs are not well understood. In cultured hippocampal neurons, increased synaptic activity induced by a glycine receptor activity blockade facilitates tubulin glutamylation and associated post-synaptic vesicular transport^12^. Here, we sought to identify environmental signals that could modify tubulin glutamylation in *C. elegans* sensory cilia (Fig. 1a). The evolutionarily conserved p38 MAPK signalling cascade is a well-studied pathway that transduces signals from various environmental stresses such as inflammatory cytokines, oxidative stress, UV irradiation, hypoxia, and ischemia^13,14^. In *C. elegans*, there are 3 orthologues of p38 MAPK (PMK-1, -2, and -3) that together form an operon on chromosome IV^15^. PMK-1 is required for innate immunity of worms and is under the control of upstream kinases NYS-1 and SEK-1^16^. As is the case with the mammalian p38 pathway, PMK-1 is also activated in response to oxidative stress in *C. elegans*^17^.

In this paper, we studied the effect of environment-induced signals on ciliary MT PTMs. We found that various environmental stimuli modify the level of tubulin glutamylation along *C. elegans* sensory cilia. Additionally, starvation activates TTLL glutamylase activity, increases anterograde IFT speeds, and suppresses of osmotic avoidance behaviour. We found evidence that environment-induced regulation of ciliary MT PTMs is conserved by showing that tubulin glutamylation is also modulated in mammalian photoreceptor cells in response to light adaptation. Furthermore, we show that the responsiveness of tubulin modification is regulated by p38 MAPK signalling, which controls the efficiency of anterograde IFT. Our results provide insights into the molecules and mechanisms governing the regulation of ciliary responsiveness to the environment and have implications for the physiological importance of reversible control of tubulin glutamylation in divergent systems.

## Results

### Tubulin glutamylation in sensory cilia is modified by the environment

To understand the role of tubulin glutamylation for physiological responses, we measured the level of glutamylation under the following environmental stimuli: starvation, low and high temperature, physical vibration, and high osmolality (Fig. 1b–g). Modification levels in amphid sensilla were quantified by immunohistochemistry using a monoclonal antibody (GT335), which specifically stains glutamylated tubulin^4^. The amount of tubulin glutamylation is normalised by the signal intensity of β-tubulin as described previously^5^. Intriguingly, all of the stimuli except vibration significantly increased the level of tubulin glutamylation in sensory cilia (33% at low temperature, *p* < 0.01; 13% at high temperature, *p* < 0.05; 38% at high osmolality, *p* < 0.01; 7% during starvation; *p* < 0.05) (Fig. 1h). Exposure of just 30 min to each stimulus is sufficient to induce an increase in glutamylation, indicating that this change is dependent on short-term processes such as protein phosphorylation and degradation, which does not depend on the lengthy process of transcription and translation.

**Figure 2:**
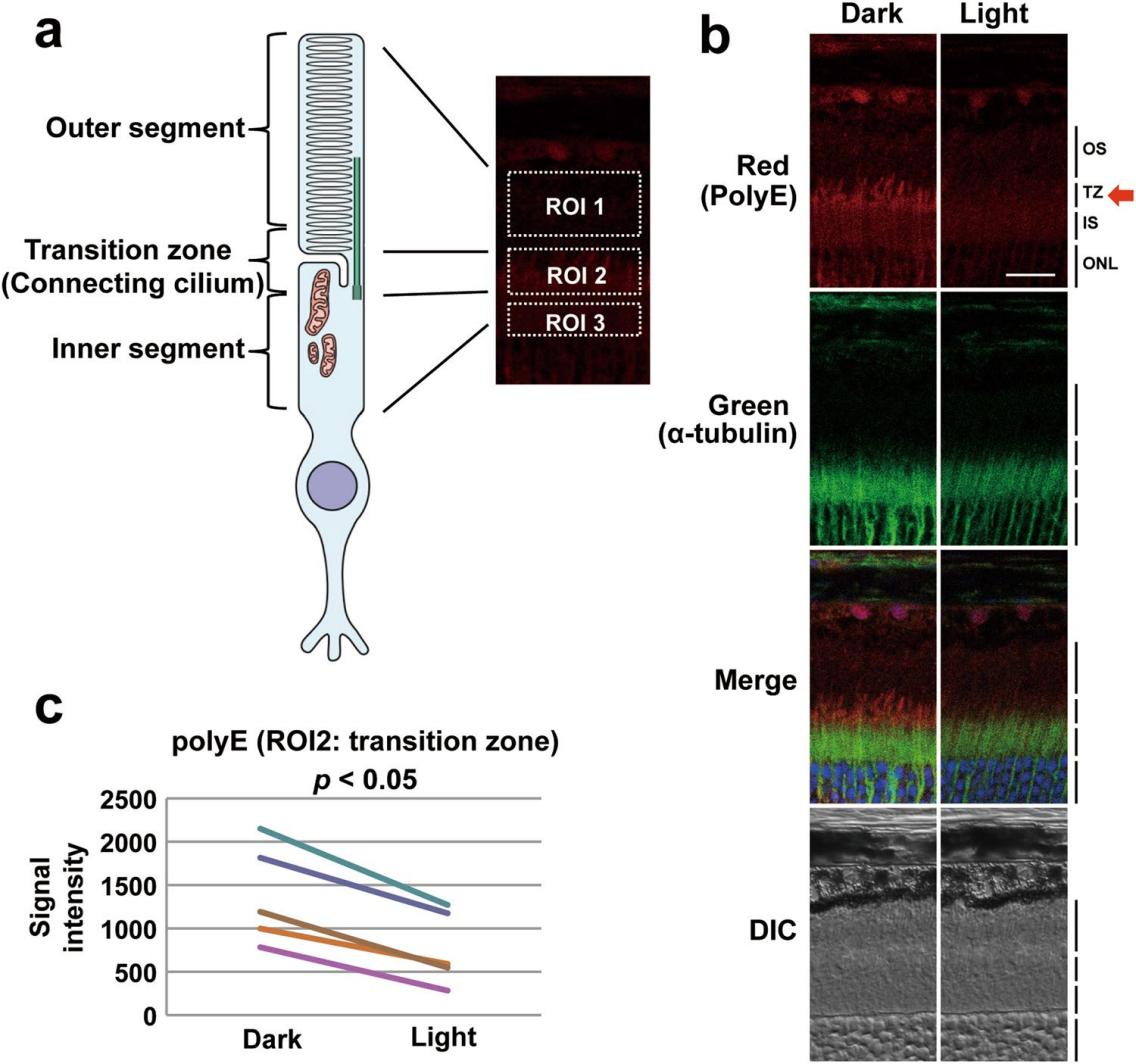
Tubulin glutamylation in mouse retina is modulated by the light exposure. (**a**) Schematic diagram of a mammalian photoreceptor cell (rod). The region was divided into three areas: outer segment, transition zone, and inner segment. (**b**) Result of immunohistochemical analysis of mouse retina using anti-polyglutamate polyclonal antibody (top, red), α-tubulin (second row, green), merged (third row), and DIC (bottom). Nuclei are shown with DAPI staining (blue). Left column: dark adaptation. Right column: light adaptation. Polyglutamate signals can be observed in connective cilia (red arrow). Tubulin signals were observed in the transition zone and inner segment. Tubulin glutamylation co-localised with tubulin in the transition zone. OS: outer segment, TZ: transition zone, IS: inner segment, ONL: outer nuclear layer. (**c**) Quantitative analysis of five independent mice. All mice have a significant reduction of tubulin glutamylation (*p* < 0.05). Scale bars = 20 μm.

To study whether the responsiveness of tubulin glutamylation to the environment is conserved in evolutionarily divergent systems, we analysed mammalian photoreceptor neurons whose connective cilia are similar to nematode sensory cilia in terms of function and structure (Fig. 2a)^18^. Using a polyclonal antibody specific for polyglutamate^19^, tubulin glutamylation was detected in the transition zone, the proximal-most part of the ciliary axoneme, referred to as the connecting cilia in photoreceptors (Fig. 2b, red arrow). We quantified and compared the level of tubulin glutamylation before and after light exposure and found that the signal intensity is significantly reduced after light exposure (47% reduction on average, *p* < 0.05; Fig. 2c). This result indicates that the environmental responsiveness of tubulin glutamylation in sensory cilia is evolutionarily conserved.

### Loss of tubulin glutamylation does not alter ciliary morphology

As we have shown previously, tubulin glutamylation in *C. elegans* sensory cilia is dependent on the tubulin glutamate ligase TTLL-4^5^. It has been shown that the level of tubulin glutamylation affects neuronal survival and morphology^6,7,10^, therefore, we analysed sensory cilia morphology in *ttll-4*(*tm3310*) mutant worms using three different methods to exclude the influence of ciliary morphology induced by the change of tubulin glutamylation. First, we used a fluorescent dye (DiI) uptake assay to indirectly assess the structure of a subset of amphid head cilia^20^ is not affected in *ttll-4* mutants, suggesting that ciliary structures are superficially normal (Supplementary Fig. S1). Second, the structure of a single cilium was directly visualised using a GFP marker (*sra-6*::GFP) expressed in two specific amphid neurons (ASH and ASI). We found that the corresponding cilia were normal in length and shape in *ttll-4* worms (Supplementary Fig. S2). Finally, we used transmission electron microscopy (TEM) to determine the ultrastructure of amphid channel cilia and found that *ttll-4* mutant worms possess normal ciliary ultrastructure, including normal MT integrity and number (Supplementary Fig. S3). Together, these three assays show that loss of tubulin glutamylation does not affect *C. elegans* cilia structure and MT integrity for the sensory neurons that were analysed.

### Responsiveness of tubulin modification is controlled by the p38 MAPK signalling pathway

To search a candidate gene responsible for the regulation of tubulin glutamylation, we first performed immunohistochemistry of GT335 for the several mutant strains that mediates the signal transduction of stress such as p38 MAPK and SAPK/JNK pathway (Supplementary Fig. S4). Therefore, the mutants of the p38 MAPK pathway gene (*nsy-1*, *sek-1*, and *pmk-1*) and those of its upstream Ca^2+^ signal transduction gene (*tax-4* and *unc-43*) showed the increase of tubulin glutamylation. On the other hand, genes of the SAPK/JNK pathway (*mek-1*, *mlk-1*, *kgb-1*, *kgb-2*, and *jnk-1*) did not show a significant difference. These results suggest that p38 MAPK pathway can mediate the control of tubulin glutamylation. Thus, we tried further analyses for p38 MAPK pathway.

**Figure 3:**
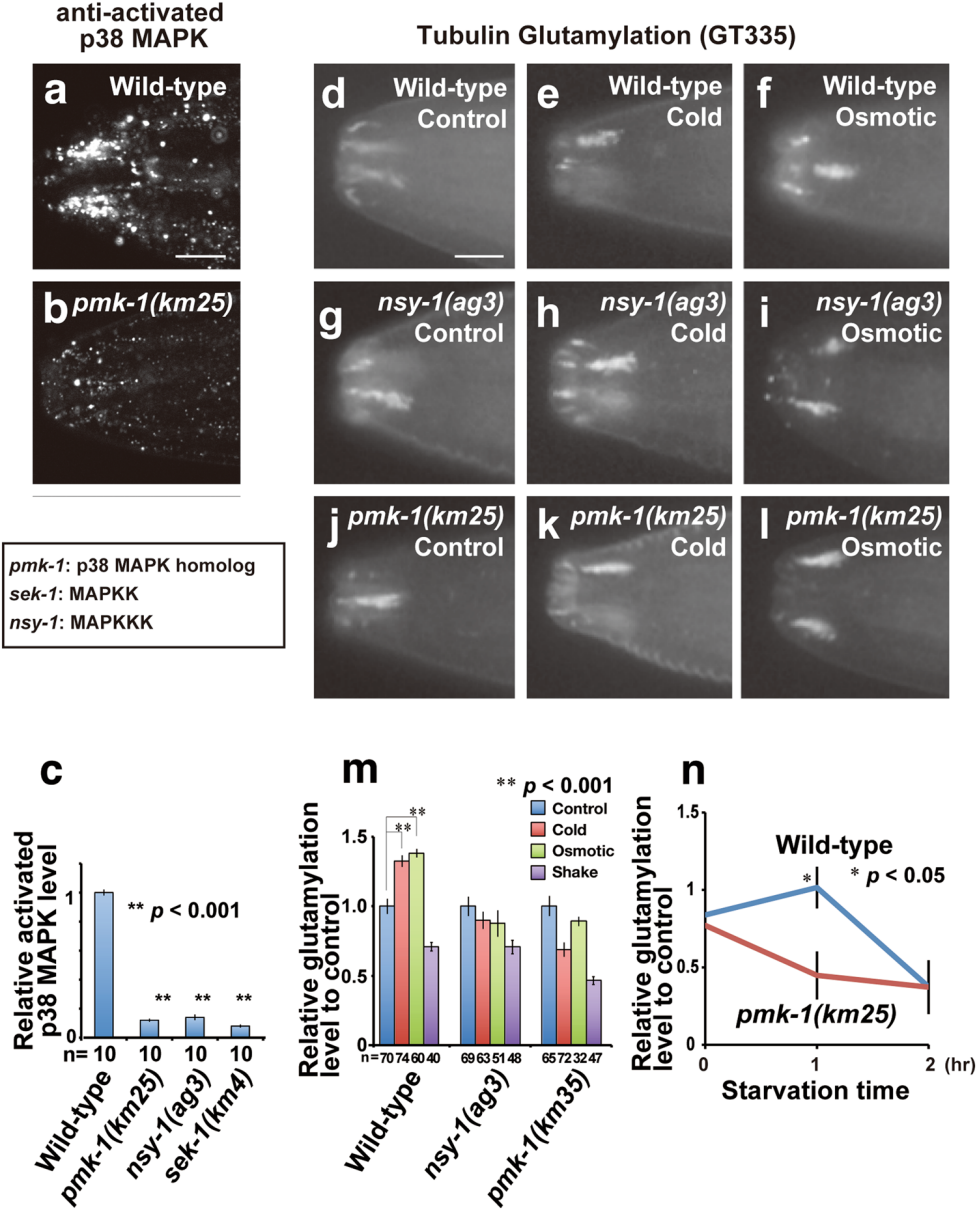
Modification of tubulin glutamylation is mediated by p38 MAPK signalling. (**a**–**c**) Results of the immunohistochemical analysis of activated p38 MAPK-specific antibody in wild-type (**a**) and a *pmk-1* mutant (**b**). Quantified result of activated p38 MAPK in wild-type and the mutants of members of the p38 MAPK signalling pathway (**c**). p38 MAPK is activated in sensory cilia in a manner dependent on the upstream kinases in the pathway. (**d**–**l**) Results of the immunohistochemical analysis of tubulin glutamylation in wild-type (**d**–**f**), *pmk-1* (**g**–**i**), and *nsy-1* (**j**–**l**) mutants under control (**d**, **e** and **j**), cold (**e**, **h** and **k**), and high osmotic (**f**, **i** and **l**) conditions. (**m**) Quantified result of tubulin glutamylation. The glutamylation levels after environmental stimuli were relative values for the control (no environmental stimuli). The enhancement of tubulin glutamylation is dependent on p38 MAPK. Y-axis is the relative glutamylation level to control (no environmental stimuli). Thus, the differences of glutamylation level seen in each mutant background were removed. (**n**) Time course analysis of tubulin glutamylation after starvation. Bars indicate mean ± S.E. All scale bars = 5 μm. An asterisk (*p < 0.05, Student’s t test) indicates significant difference as compared with the signal intensity before starvation in wild-type (**n**). Double asterisks (**p < 0.001, Student’s t test) indicate significant difference as compared with the wild-type (**c** and **m**). Numbers of animals scored are indicated (**c** and **m**, bottom).

To determine if the p38 MAPK pathway contributes to modulation of ciliary MT PTM in *C. elegans*, we investigated p38 MAPK activity using an antibody specific for activated p38 MAPK. In wild-type worms, high levels of activated p38 MAPK were detected around sensory cilia (Fig. 3a). This activation disappeared in a *pmk-1* null allele^17^ (Fig. 3b), indicating that p38 activation was PMK-1-dependent. Additionally, worms deficient for *sek-1* and *nsy-1*, which respectively encode upstream MAPKK and MAPKKK regulators of PMK-1^21,22^, also display a consistent loss of activated p38 MAPK in cilia, indicating that PMK-1 is activated in sensory cilia under the control of these upstream kinases (Fig. 3c).

Double staining of GT335 and activated p38 MAPK with or without environmental stress indicates that not only GT335 but also activated p38 MAPK signal is reasonably increased by high osmolality (Supplementary Fig. S5). The activated p38 MAPK is expressed in the much wider area around the amphid, especially after the stress exposure (Supplementary Fig. S5d,f). In terms of the expressions inside amphids, the punctate expressions of activated p38 MAPK are very restrictedly localised in comparison to the broad expression of GT335 (Supplementary Fig. S5c,f).

Next, we assessed if the enhanced ciliary tubulin glutamylation levels we observed following exposure to altered environmental conditions (Fig. 1) is dependent on the p38 MAPK pathway. As shown in Fig. 3d,g,j, and Supplementary Fig. S4, mutants of the p38 MAPK pathway (*pmk-1* and *nsy-1*) showed higher tubulin glutamylation levels even without the exposure of environmental stimuli. In wild-type worms, the exposure to cold temperatures or high osmolality increased tubulin glutamylation (Figs 1b–h, 3d–f,m). In contrast, the mutants of *pmk-1* and *nsy-1* genes did not show an additional enhancement in response to environmental stimuli. (Fig. 3g–l,m). These results indicate that stress-induced increase of glutamylated tubulin in sensory cilia is dependent on the p38 MAPK pathway. A time-course analysis of starvation-induced enhancement has revealed that a significant increase was observed after 1 hr of starvation (Fig. 3n). These results mean that dietary status has similar effects to tubulin glutamylation as well as those observed by the exposure to excess environmental stimuli.

### Phosphorylation of Thr^446^ in TTLL-4 enhances ciliary tubulin glutamylation

**Figure 4:**
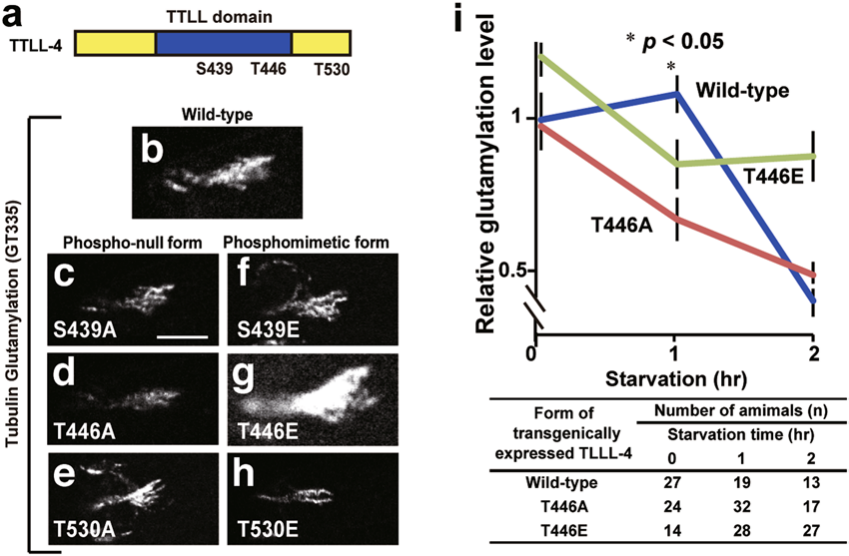
Tubulin glutamylation is regulated by the phosphorylation of tubulin glutamate ligase TTLL-4. (**a**) Schematic structure of TTLL-4. Blue area marks the TTLL domain, which is essential for the enzymatic activity. Ser or Thr residues that can be putatively phosphorylated by MAPK are indicated. (**b**–**h**) Tubulin glutamylation induced by exogenous expression of wild-type and mutant forms of TTLL-4. (**i**) Time course analysis of tubulin glutamylation after starvation. Wild-type: *ttll-4* mutant exogenously expressing wild-type TTLL-4; T446A: *ttll-4* mutant exogenously expressing phosphor-null TTLL-4; T446E: *ttll-4* mutant exogenously expressing phosphomimetic TTLL-4. An asterisk (*p < 0.05, Student’s t test) indicates significant difference as compared with the signal intensity before starvation in wild-type (**i**). Numbers of animals measured are indicated (bottom). Bars indicate mean ± S.E. Scale bar = 5 μm.

We have previously shown that TTLL-4 protein is localised in amphid^5^, which prompted us to investigate if TTLL-4 regulation is under the control of the p38 MAPK pathway. TTLL-4 has three amino acid residues (Ser^439^, Thr^446^, and Thr^530^) that are candidates for phosphorylation and regulation by MAPK (Fig. 4a and Supplementary Fig. S6). To determine if these residues are required for TTLL-4 activity, we mutated the amino acids to either a phospho-null residue (alanine) or a phosphomimetic residue (glutamate). The resulting TTLL-4 variants were transgenically expressed in the sensory neurons of *ttll-4*-deficient worms to assess their ability to induce ciliary tubulin glutamylation. Under normal growing conditions, most worms expressing the phospho-null and phosphomimetic TTLL-4 variants had similar levels of ciliary tubulin glutamylation compared to worms expressing the wild-type TTLL-4 transgene (Fig. 4b–f,h). However, worms expressing the TTLL-4(T446E) transgene had increased ciliary tubulin glutamylation levels compared to the wild-type control (Fig. 4b,g), indicating that phosphorylation of Thr^446^ enhances TTLL-4 activity. Since worms expressing the non-phosphorylated form of TTLL-4 at amino acid 446 (T446A) maintained a normal level of activity, this residue was determined to be non-essential for baseline activity of TTLL-4 (Fig. 4b,d).

Next, *ttll-4*-deficient worms expressing wild-type and mutant variants (T446A and T446E) of TTLL-4 were exposed to starvation conditions (Fig. 4i). Similar to wild-type worms (Fig. 3n), *ttll-4* mutant worms expressing the wild-type TTLL-4 transgene had increased ciliary tubulin glutamylation (Fig. 4i). In contrast, worms expressing TTLL-4(T446A) did not have an increase in glutamylation, suggesting that phosphorylation of Thr^446^ is required for starvation-induced increase of ciliary tubulin glutamylation. Worms expressing TTLL-4(T446E), whose modification level was higher than that of wild-type before starvation, did not have any additional enhancement; rather, we found a reduction of the signal intensity. Similarly, the increase of glutamylation seen in the worms with wild-type TTLL-4 is temporally regulated: the reduction of signal was detected 2 hr after starvation. These results indicate that phosphorylation of Thr^446^ is required for the temporal activation of TTLL-4.

### Tubulin glutamylation controls the efficiency of IFT in sensory cilia

We assessed the effects of *C. elegans* tubulin modification on IFT. In amphid and phasmid channel cilia, two kinesin-2 motors (heterotrimeric kinesin-II and homodimeric OSM-3) cooperate to drive anterograde IFT along the proximal ciliary region, which is the middle segment (MS) that contains doublet MTs. Anterograde transport along the distal ciliary portion, which is the distal segment (DS) containing singlet A-tubules, is driven by OSM-3 alone^23–26^. The result of this arrangement is that anterograde IFT motors move at roughly 0.7 μm/sec along the MS, and at roughly 1.2 μm/sec along the DS^23–25^. Using an OSM-6/IFT52::GFP marker for IFT^23–26^, we first analysed the speed of anterograde IFT motors along the phasmid cilia of *ttll-4* (no tubulin modification) mutants. Compared to wild-type worms, we observed a modest reduction of MS and DS IFT rates in *ttll-4* mutants (Supplementary Fig. S7). These results suggest that there is a positive correlation between the IFT speed of particles and the level of tubulin glutamylation in the cilia.

**Figure 5:**
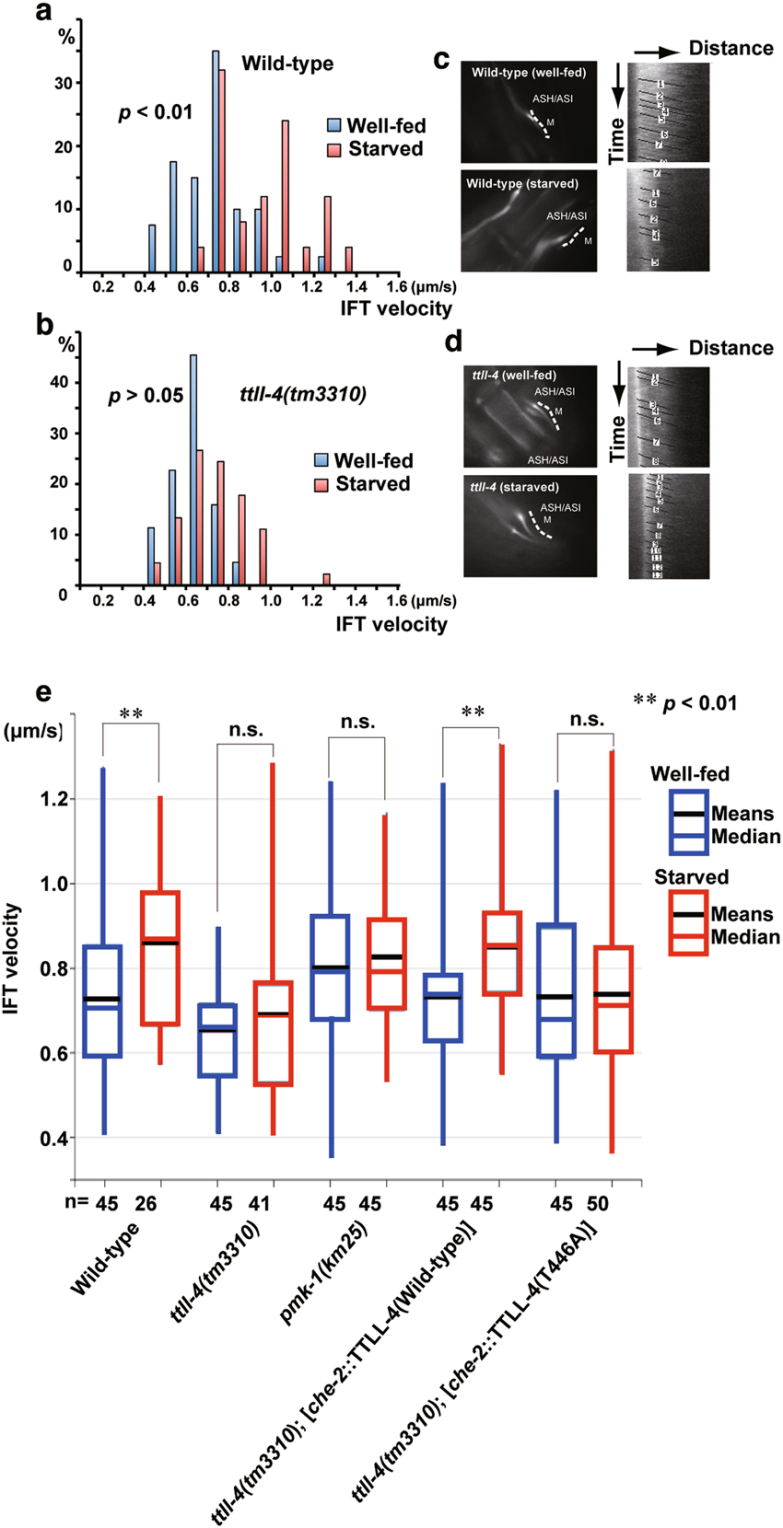
IFT is enhanced by starvation in a TTLL-4-dependent manner. (**a**–**d**) Result of IFT velocity analysis. (**a**,**b**) Motility of KAP-1::GFP within cilia of wild-type animals (upper) and *ttll-4* mutants (lower). Left: histogram of transport velocities. The number of particles moving at the indicated ranges of velocity is shown. Right: fluorescence micrographs with corresponding schematic showing lines used to generate kymographs along the MS. Kymographs and corresponding lines show that motility after starvation is faster than that of the untreated condition. (**e**) Box and whisker distribution plots of IFT velocities in well-fed (blue) and starved (red) worms. Boxes indicate quartiles. Whiskers indicate minimum and maximum. Black lines in each box indicate means and the lines of the same colour in each box indicate median. The enhancement of IFT velocity is *ttll-4*- and *pmk-1*-dependent. The phenotype of *ttll-4* mutants is rescued by exogenous expression of wild-type TTLL-4. However, a phospho-null form of TTLL-4 cannot rescue the *ttll-4* phenotypes. Velocities derived from multiple kymographs (in bracket) and the number of particles measured were as follows: well-fed wild-type: 45 (10), starved: 26 (5), well-fed *ttll-4*: 45 (8), starved *ttll-4*: 41(5), well-fed *ttll-4* + *che-2::*TTLL-4(Wild-type): 45(10), starved *ttll-4* + *che-2::*TTLL-4(Wild-type): 45(10), well-fed *ttll-4* + *che-2::*TTLL-4(T446A): 45(10), starved *ttll-4* + *che-2::*TTLL-4(T446A): 45(10), well-fed *pmk-1*: 45(5), and starved *pmk-1*: 50(10). Double asterisks (**p < 0.01, Mann-Whitney’s U test compared with well-fed worms) indicate significant difference as compared with the velocities between well-fed and starved animals (**e**). Numbers of animals measured are indicated (e, bottom). n.s.: not significant difference between well-fed and starved worms.

Next, we tested if starvation-induced glutamylation of ciliary MTs affects IFT. For these experiments, we used GFP-tagged KAP-1 (a kinesin-II subunit) under the control of the *sra-6* promoter, which drives expression in only two amphid channel neurons (ASH and ASI). Like our findings for OSM-6::GFP, the anterograde speeds of KAP-1-marked IFT motors was reduced in the MS of *ttll-4* mutant ASH and ASI cilia compared to wild-type controls (Fig. 5a–d; Table 1; Supplementary Fig. S7). One-hour starvation increased KAP-1 speeds in wild-type worms, but not in *ttll-4* mutants (Fig. 5b,e). Expression of a wild-type TTLL-4 transgene in the *ttll-4* mutant rescued the starvation-induced IFT defect, whereas this defect was not rescued in *ttll-4* worms expressing a phospho-null TTLL-4 variant at residue 446 (T446A) (Fig. 5e; Table 1; Supplementary Fig. S8). Importantly, the velocities of IFT in well-fed *ttll-4*-deficient animals were rescued by both a wild-type and a phospho-null TTLL-4, suggesting that phosphorylation of Thr^446^ is essential only for starvation-induced enhancement of IFT but not for the maintenance of basic level of IFT (Fig. 5e; Table 1; Supplementary Fig. S8).

**Table 1:** IFT velocities of KAP-1 protein along middle segments of amphid in well-fed and starved worms.

Strain	Condition	n/N	Mean (μm/s)	Median (μm/s)	Mann-Whitney’s U test
Wild-type	Well-fed	45/10	0.73 ± 0.18	0.70	
	Starved	26/5	0.86 ± 0.19	0.87	*p* < 0.01
*ttll-4*(*tm3310*)	Well-fed	45/8	0.65 ± 0.12	0.66	
	Starved	41/5	0.69 ± 0.18	0.69	n.s.
*pmk-1*(*km25*)	Well-fed	45/10	0.80 ± 0.17	0.79	
	Starved	45/10	0.83 ± 0.15	0.79	n.s.
*ttll-4*(*tm3310*); [*che-2*::TTLL-4(Wild-type)]	Well-fed	45/10	0.73 ± 0.15	0.74	
	Starved	45/10	0.85 ± 0.16	0.85	*p* < 0.01
*ttll-4*(*tm3310*); [*che-2*::TTLL-4(T446A)]	Well-fed	45/5	0.73 ± 0.19	0.68	
	Starved	50/10	0.74 ± 0.20	0.71	n.s.

Data are expressed mean ± SD, median, and the number of particles (n) and number of worms analysed (N). Mann-Whitney’s U test was preformed to compare well-fed and starved worms for each strain. n.s.: not significant.

### TTLL-4 is required for the starvation-induced modification of sensory behavior

**Figure 6:**
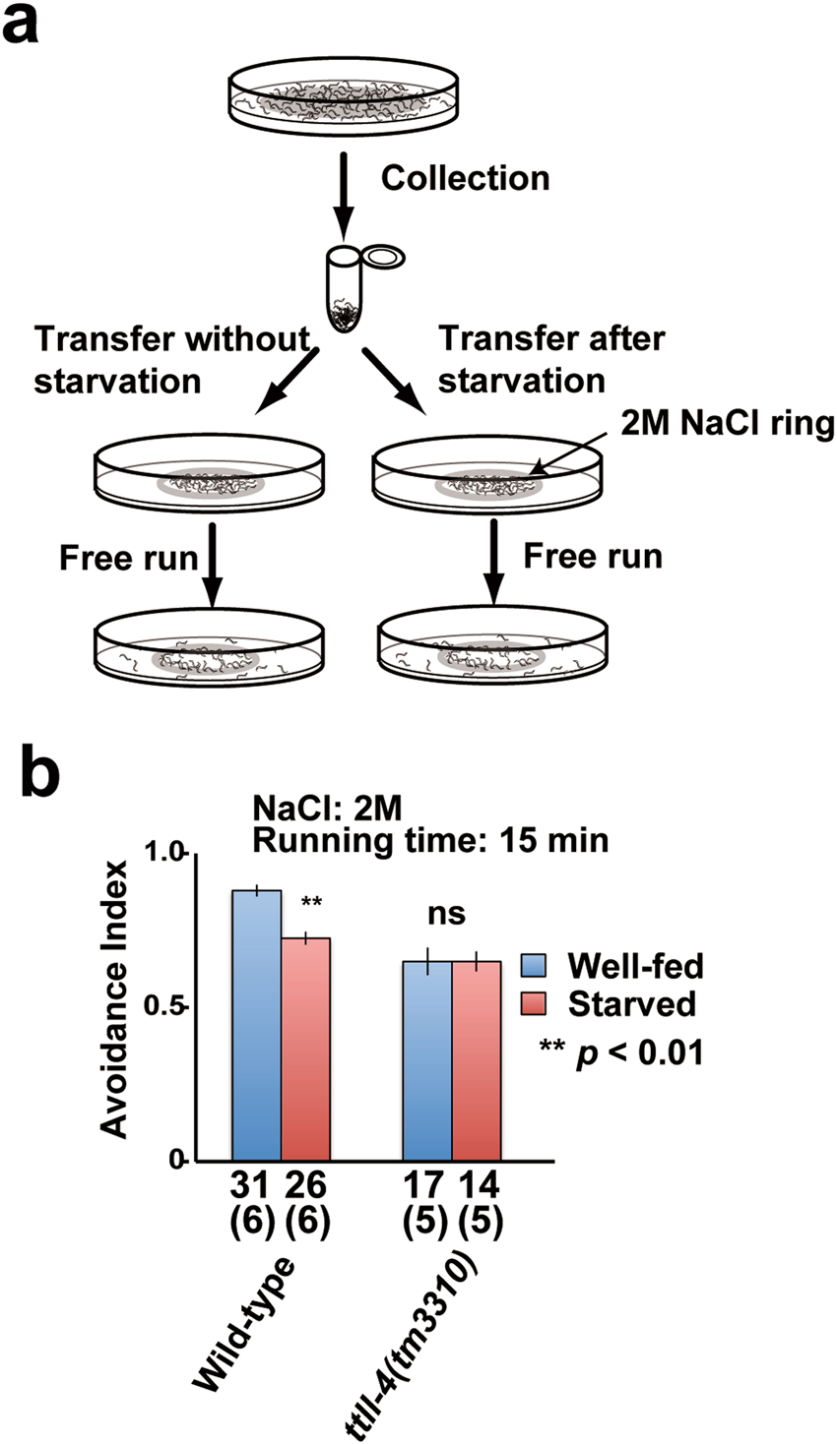
Starvation-induced attenuation of osmotic avoidance behaviour is dependent on tubulin glutamylation. (**a**) Schematic diagram of the sensitised osmotic avoidance assay. (**b**) TTLL-4-dependent suppression of avoidance behaviour is induced by starvation. Numbers of assay plates measured are indicated. Numbers in brackets represent the number of independent experiments. Double asterisk (^**^p < 0.01, Student’s t test) indicates significant difference as compared with the avoidance index after starvation in wild-type. Bars indicate mean ± S.E.

Finally, we addressed the possibility that starvation-induced increase of tubulin glutamylation modifies worm sensory behaviour. The avoidance behaviour towards high osmolality is one of the best-characterised sensory behaviours of *C. elegans*^27^. Since *ttll-4*-deficient worms showed subtle defects of avoidance behaviour towards high osmolality^28^, the assay was sensitized using as low as 2 M NaCl solutions (Supplementary Fig. S9). To study the environmental effects for osmotic avoidance, we analysed the influence of starvation (Fig. 6a) and found that the avoidance index is lowered by 17% due to starvation (from 0.87 to 0.72, *p* < 0.01; Fig. 6b left). This result means that starvation reduces the sensitivity to high osmolality. A *ttll-4* gene essential for tubulin glutamylation in sensory cilia is required for various sensory behaviours (Supplementary Fig. S9). To elucidate the relationship between the tubulin glutamylation and behavioural modification in response to environmental stimuli, we measured the effect of this gene on the responsiveness to starvation. Importantly, this starvation-induced suppression was not observed in *ttll-4*-deficient worms (Fig. 6b right). This result indicates that starvation-induced modification of sensory behaviours is dependent on tubulin glutamylation in sensory cilia.

## Discussion

We previously showed reversible enzymatic regulation of tubulin glutamylation is conserved in *C. elegans* and mammals^5^. In this paper, we studied the responsiveness of tubulin glutamylation to the environment in two organisms. In *C. elegans* sensory cilia, tubulin glutamylation increases in response to specific signals from the environment such as temperature, osmolality, and starvation (Fig. 1b–h). On the other hand, light adaptation in the mouse retina decreases the level of tubulin glutamylation in the connecting cilia that bridge the inner and outer segments of the photoreceptor neuron (Fig. 2b,c). One possible explanation for this difference is that each animal adopts different signalling pathways to couple environmental influences with tubulin glutamylation. We have shown that the p38 MAPK pathway mediates responsiveness in *C. elegans*. However, the rhodopsin (G protein-coupled receptor [GPCR])-transducin (G protein) pathway has an essential role in visual phototransduction in mammals^29^. Visual arrestin, which plays a critical role in the signalling of GPCRs in photoreceptor cells, binds the negatively charged carboxyl-terminus of MTs^30^. Since visual arrestin moves from the inner to outer segments of a photoreceptor cell during light adaptation, the reduction of tubulin glutamylation in the connecting cilia between the segments could facilitate this protein movement. p38 MAPK is also activated in response to blue light exposure in cultured photoreceptor cells. However, p38 MAPK activation in this context mainly serves to protect cells against the toxicity of blue light^14,31^. In summary, the environment-induced flexibility of tubulin glutamylation in cilia is conserved in different organisms, suggesting its physiological importance for regulating sensory systems. Recently, it has reported that a loss of glutamylation in a photoreceptor-specific form of RPGR (a GTPase regulator), which is located in the connecting cilia, causes photoreceptor degeneration^32^. This result suggests that not only tubulin glutamylation but also the glutamylation of other substrates is important for the maintenance of the photoreceptors.

There are some reports that PMK-1 is required for the stress response of both hypoxia and oxidative conditions in *C. elegans*^17,33^. In addition, PMK-1 is also required for extracellular vesicle biogenesis from sensory cilia^34^. This is consistent with our result that PMK-1 is activated in sensory cilia (Fig. 3a–c). In these cases, however, PMK-1 is activated by a non-canonical pathway^16,30^. On the other hand, our present study indicates that p38 MAPK is activated under the control of NSY-1 and SEK-1, which is similar to the innate immune system^16^.

Amino acid exchange analyses have revealed that the putative MAPK phosphorylation site Thr^446^ is required for starvation-induced activation of TTLL-4 (Fig. 4). Interestingly, tubulin glutamylation of wild-type worms began to decrease after 1 to 2 hr of starvation. We hypothesized that this is caused other starvation-induced pathway(s) that suppress tubulin glutamylation independently of Thr^446^ of TTLL-4. Worms with non-phosphorylated and phosphomimetic TTLL-4 had a decrease in glutamylation just after starvation (Fig. 4i). Worms with the phosphomimetic TTLL-4 showed higher tubulin glutamylation than wild-type in the background before any treatments, however, additional enhancement does not occur after starvation. We speculate that it is because Thr^446^-dependent activation is already “saturated” by the enhancement caused by amino acid exchange. These results suggest that the starvation-induced signal decrease began much earlier even in wild-type worms, however, this decrease in the background could be “masked” by Thr^446^-dependent temporary increase.

According to the 3D-structure of tubulin tyrosine ligase (TTL)^35^, the putative phosphorylation site essential for the starvation-dependent enhancement is located in the loop area of the TTLL domain, suggesting that phosphorylation of this target amino acid could alter the structure of the active site. Amino acid sequence comparison (Supplementary Fig. S6) has revealed that this phosphorylation site is evolutionarily conserved in human TTLL4. Therefore, the mechanism for MAPK-mediated phosphorylation and activation of TTLL-4 could be conserved in mammals. This specific Ser or Thr exists in only in two members of the TTLL family: TTLL-4 and TTLL8 (a tubulin glycine ligase) (Supplementary Fig. S6).

Starved worms undergo several stages of starvation. Long-term starvation can induce many developmental and physiological changes followed by biosynthetic activity, such as either progressing from larvae through to the normal reproductive cycle or to the dauer stage^36^. Still, even short-term starvation (<3 hr) is enough to modify cellular physiology of neurons^37^. Our current study has shown that IFT in the MS of cilia is facilitated by starvation and this effect is regulated by the phosphorylation-dependent activation of tubulin glutamylase TTLL-4. This is consistent with the result that tubulin glutamylation specifically occurs in the MT doublet region (MS)^5,38^. Recently, Prevo *et al*. reported that kinesin-II drives entry into worm cilia and then gradually hands over cargo to OSM-3^39^. This means that velocity changes induced by starvation were also influenced by the interaction between two different motor proteins as well as the glutamylation enhancement along MT.

There are several reports that short-term starvation (<1 hr) can modify sensory behaviours^40,41^. For example, learning to associate cultured conditions with starvation can occur in a short time. Worms can store the memory of the temperature at which they endured starvation and will then avoid those specific conditions^42–44^. Recently, it has been reported that the memory of temperature is stored in sensory neurons through a CaM kinase I/IV-Raf pathway^43^. Similarly, chemotaxis to NaCl is modulated by associative learning between specific salt concentrations and starvation^41,45^. Insulin/PI3 kinase pathway and CASY-1 (an orthologue of calsyntenins) is essential for salt chemotaxis learning^46^. In the present study, we also analysed the modulation of behaviours in short-term starvation (<1 hr), which is too brief for gene expression to be initiated but is long enough for signal transduction or PTM to occur. We focused on tubulin glutamylation performed by a tubulin glutamylase TTLL-4, which is predominantly localised in sensory cilia and essential for tubulin glutamylation along axonemal MTs^5^. We found that avoidance behaviour to high osmolality is attenuated by starvation, which is a novel TTLL-4-dependent modulation of sensory behaviour in response to environmental cues. We speculate that this temporal suppression of avoidance from hazardous environments is essential to encourage a starved animal to enter undesirable environments in the search for food. Our results reveal a novel and important regulatory mechanism for intracellular transportation and organelles that is used to modulate chemosensory behaviour.

## Methods

### Worm strains

Nematodes were grown at 20 °C using standard methods described by Brenner^47^ unless otherwise noted. The following mutant strains were used in this study: YT315: *ttll-4*(*tm3310*) III, YT426: *ccpp-6*(*ok382*) II, KU25: *pmk-1*(*km25*) IV, AU3: *nsy-1*(*ag3*) II, and KU4: *sek-1*(*km4*) X.

### Transgenic strains

The host strain was wild-type N2. The pRF4 plasmid (Rol) or *sur-5*::mCherryNLS were used as a coinjection marker. The quantity of all test DNAs was 30–100 ng/μl. To compare the same transgenic array under the different genetic background, the arrays were transferred to the other strains by genetic crossing. The transgenic strains used in this paper is as follows,

YT1585: *ttll-4*(*tm3310*)III; *tzEx1580*[*che-2*::TTLL-4::mCherry; *rol-6*(+)],

YT1590: *ttll-4*(*tm3310*)III; *tzEx1590*[*che-2*::TTLL-4(T446A)::mCherry; *rol-6*(+)],

YT1581: *ttll-4*(*tm3310*)III; *tzEx1581*[*che-2*::TTLL-4(T530A)::mCherry; *rol-6*(+)],

YT1623: *ttll-4*(*tm3310*)III; *tzEx1623*[*che-2*::TTLL-4(S439E)::mCherry; *rol-6*(+)],

YT1636: *ttll-4*(*tm3310*)III; *tzEx1636*[*che-2*::TTLL-4(T446E)::mCherry *rol-6*(+)],

YT1603: *ttll-4*(*tm3310*)III; *tzEx1603*[*che-2*::TTLL-4(T530E)::mCherry; *rol-6*(+)],

YT384: *tzEx384*[*sra-6*::KAP-1::GFP *rol-6*(+)],

YT528: *tzEx384*[*sra-6*::KAP-1::GFP *rol-6*(+)]; *ccpp-6*(*ok382*)II,

YT505: *tzEx384*[*sra-6*::KAP-1::GFP *rol-6*(+)]; *ttll-4*(*tm3310*)III,

YT525: *tzEx384*[*sra-6*::KAP-1::GFP *rol-6*(+)]; *pmk-1*(*km20*)IV,

YT529: *ttll-4*(*tm3310*)III; *tzEx384*[*sra-6*::KAP-1::GFP *rol-6*(+)]; tzEx1636[*che-2*::TTLL-4::mCherry *sur-5*::mCherryNLS],

YT530: *ttll-4*(*tm3310*)III; *tzEx384*[*sra-6*::KAP-1::GFP *rol-6*(+)]; tzEx1636[*che-2*::TTLL-4(T446A)::mCherry *sur-5*::mCherryNLS].

### Immunohistochemistry of worms

Immunohistochemistry was performed as described previously^5^. The animals were fixed in modified Bouin’s fixative for 30 min and immediately frozen in liquid nitrogen. Antibody incubation was performed overnight at 4 °C in a blocking solution. GT335 antibody (kindly provided by Dr. B. Eddé) was diluted to a ratio of 1:2.5 × 10^6^ (for signal quantification, as shown in Figs 1b–g, 3d–l, and 4b–h) before incubation. The anti-β-tubulin polyclonal antibody (MBL, Nagoya, Japan) was diluted 1:200. The anti-active p38 polyclonal antibody (Promega, Fitchburg, WI, USA) was diluted 1:200. After washing with antibody buffer, the worms were incubated overnight in 1:500 Alexa Fluor 488- or 594-conjugated anti-mouse or anti-rabbit secondary antibody, respectively (Molecular Probes, Eugene, OR, USA). After washing with antibody buffer, samples were analysed by an upright fluorescent microscopy AX80 (Olympus, Tokyo, Japan) or a confocal microscopy FV1000 (Olympus, Tokyo, Japan).

### Quantification of the glutamylated tubulin

Quantification of glutamylated tubulin was performed as described previously^5^ with slight modifications. After GT335 and β-tubulin signal intensities were measured in sensory cilia from the same optical slice, the subtraction of background signals in non-ciliary regions were performed. A GT335/β-tubulin signal intensity ratio was calculated for each image. The relative intensity of each worm strain was calculated by the average of GT335/β-tubulin normalised by that of wild-type, and statistically analysed by Microsoft Excel (Microsoft, Redmond, WA, USA) and StatPlus (AnalystSoft, Walnut, CA, USA).

### DNA construction

The vector *che-2*::TTLL-4::mCherry was constructed as described previously^5^. PCR-based nucleotide exchanges were performed to make amino acid substitutions at S439A, S439E, T446A, T446E, S530A, and S530E. The *sur-5*::mCherryNLS maker gene was a gift from Dr. K. Fujisawa and Dr. J. G. Culotti. The *sra-6*::KAP-1::GFP was a gift from Dr. P. Sengupta.

### IFT and dendritic transport analyses

Time-lapse imaging and analyses were performed as described by Fujiwawa *et al*.^48^. Time-lapse images were obtained with the Olympus IX71 microscope equipped with 100×, 1.4 N.A. objective and ORCA-ER digital camera (Hamamatsu Photonics, Hamamatsu, Shizuoka, Japan) with Uniblitz VMM-D1 shutter unit (Vincent Associates, Rochester, NY, USA). All images were collected at 1 frame/0.5 sec intervals, and at 100–400 ms exposure time for 1 min duration, with adult hermaphrodite anaesthetised with 10 mM levamisole.

### Osmotic avoidance assay

Osmotic avoidance assays were carried out as described^27,49^ with minor modifications. In this paper, we used 2 M NaCl solutions to detect subtle differences after comparing various concentrations of NaCl and glycerol. Avoidance indexes were calculated by dividing the number of worms inside the circle of high osmolality by the total number of worms.

### Stress treatment of worms

Worms were incubated under stressed conditions as follows. All worm-washing steps were performed with a wash buffer (0.02% gelatin, and 25 mM pH 6.0 potassium phosphate). For high or low temperature conditions, plates were incubated for 30 min in a 4 °C or 37 °C incubator. After incubation, animals were washed and immediately used for further analyses. For the starvation assays, animals were collected into siliconised 2.0 ml microcentrifuge tubes by washing the plates. After two washing steps, worms are incubated in wash buffer for 30 min with slow rotation in the microcentrifuge tubes (<30 rpm) using the RT50 rotator (TAITEC, Koshigaya, Saitama Japan). For the high osmolality conditions, worms were washed and then soaked in 0.6 M glycerol solution with slow rotation in microcentrifuge tubes. After incubation, worms were washed with wash buffer and immediately used for further analyses. Finally, for the vibration assays, plates were fixed on a gyratory shaker and shaken fiercely (>180 rpm). After vibration, animals were washed and used for further analyses.

### Murine experiments

The wild-type C57BL/6 J mouse strain was used. All experimental procedures were approved by the Institutional Animal Care and Use Committees of Hamamatsu University, School of Medicine, and are in accordance with the Japanese Fundamental Guidelines for Proper Conduct of Animal Experiment and Related Activities in Academic Research Institutions under the jurisdiction of the Ministry of Education, Culture, Sports, Science and Technology.

### Light-dark adaptation

Mice were kept in a dark room overnight and their eyes were dissected in the dark with infrared goggles (NV-G1, Japan Medical services, Tokyo, Japan). Dissected eyes were placed in DMEM. One eye from each pair was kept in the dark and the other was illuminated. After 30 min of incubation, all pairs of eyes were fixed with 4% PFA in 0.1 M phosphate buffer (PB) (pH 7.2) for 6 hr during the fixation, dark-adapted eyes were shielded from light. The eyes were then cryoprotected with 10% and then 20% sucrose/PBS and embedded in optimal cutting temperature compound (SAKURA Finetek, Tokyo, Japan). Sections were cut in the plane parallel to the optic nerve with a cryostat (CM1950 Cryostat, Leica Microsystems, Wetzlar, Germany). For immunostaining, sections within 300 μm from the meridian plane were used. Each pair of eyes from the same mouse (where one is dark-adapted and the other is light-adapted) were put on the same glass slide and stained under identical conditions and stored at −80 °C until use.

### Immunohistochemistry of mice

Sections were air-dried and fixed with 4% PFA/0.1 M PB (pH 7.2) for 30 min at room temperature to prevent detachment from the glass slide. After washing with PBS three times, sections were treated with freshly prepared 0.1% NaBH_4_/PBS three additional times for 10 min each time. The sections were rinsed with PBS several times and treated with 1% Triton X-100/PBS for 30 min. They were then treated with blocking solution (5% goat serum, 0.1% Triton X-100/PBS) following several rinses with PBS. The sections were sequentially treated with primary antibody (polyE, 1:500, in blocking solution) overnight at 4 °C, secondary antibody (Alexa 568, 1:500, in blocking solution) for 1 hr at room temperature, and the blocking solution containing DAPI (1:10,000) and anti-α-tubulin-FITC (1:500) for 10 min at room temperature. Finally, the samples were mounted with 90% glycerol/PBS. Between each step, sections were washed with PBS three times for 10 min per wash. The sections were observed with a confocal microscope (FV1000, Olympus, Tokyo, Japan).

### Electron microscopy

Transmission electron microscopy of amphid channel cilia was performed on processed day 1 adult worms as previously described^50^.

### Data Availability

The datasets generated and analysed, and full sets of results obtained during the current study are available from the corresponding author on reasonable request.

## Electronic supplementary material

Supplementary information
