# Supplementary material for: Environmental responsiveness of tubulin glutamylation in sensory cilia is regulated by the p38 MAPK pathway
Source: Sci Rep. 2018 May 30;8:8392. doi: 10.1038/s41598-018-26694-w (PMC5976657; doi:10.1038/s41598-018-26694-w)

Supplementary information

Environmental responsiveness of tubulin glutamylation in sensory cilia is regulated by the p38 MAPK pathway

Yoshishige Kimura, Koji Tsutsumi, Alu Konno, Koji Ikegami, Saira Hameed, Tomomi Kaneko, Oktay Ismail Kaplan, Takayuki Teramoto, Manabi Fujiwara, Takeshi Ishihara, Oliver E. Blacque & Mitsutoshi Setou

Supplementary Methods

Supplementary References

Supplementary Figure Legends S1-S9

Supplementary Figures S1-S9

Supplementary Methods

Strains. The following worm strains were used for the supplementary data:

Mutant strains:

AU3: *nsy-1(ag3)* II, AU4: s*ek-1(km4)* X, CB408: *unc-43(e408)* IV, KB7: *kgb-1(um3) kgb-2(km16)*IV, KF171: *mek-1(ks54)* X, KU25: *pmk-1(km25)* IV, MT6308: *eat-4(ky5)*III, PR678: *tax-4(p678)* III, RB1908: *mlk-1(ok2471)*V, VC8: *jnk-1(gk7)* IV, and YT315: *ttll-4(tm3310)* III.

Transgenic strains:

YT338: *ttll-4(tm3310)* III; *mnIs17*[OSM-6::GFP; *unc-36*(+)],

YT422: *ttll-4(tm3310)* III; *tzEx*[*sra-6*::GFP],

YT501: *tzEx*[*sra-6*::GFP],

YT1580: *ttll-4*(*tm3310*) III; t*zEx*[*che-2*::TTLL-4::mCherry; *rol-6*(+)]

YT1590: *ttll-4*(*tm3310*) III; *tzEx*[*che-2*::TTLL-4(T446A)::mCherry; *rol-6*(+)],

SP2101: *ncl-1(e1865) unc-36(e251)* III; *osm-6(p811)* V; *mnIs17*[OSM-6::GFP; *unc-36*(+)],

Behavioural analysis. Detailed protocols of behavioural analyses were described previously[1]. Nose touch assay was performed by laying a hair on the surface of the plate in front of the animal. Normal animals that responded with reverse movement were counted. Chemotaxis assays for NaCl were performed as described previously[2]. Collected worms were conditioned with or without NaCl. Osmotic avoidance assays were performed as described previously[3,4] with a slight modification: we compared different concentrations of NaCl to detect the subtle differences of *ttll-4* mutants.

Dye-filling assay. Dye-filling assays were done as described previously using DiI (10

mg/ml)[5]. After washing with M9, worms were observed with an FV1000 confocal microscope (Olympus, Tokyo, Japan).

Transmission electron microscopy. TEM of amphid channel cilia was carried out as described previously[5].

Visualising sensory cilia of *C. elegans*. Sensory cilia of ASI and ASH in amphids were visualised with *sra-6*::GFP as a marker and observed by FV1000 (Olympus, Tokyo, Japan).

Amino acid alignment. Amino acid comparisons of *C. elegans* proteins and the human TTLL family were performed using GENETYX-Mac Version 18 (GENETYX, Tokyo, Japan).

Supplementary figure legends

Supplementary figure S1. Dye-filling analysis for *ttll-4* mutants. Amphid neurons were visualised by soaking in DiI solution. Wild-type (a), *ttll-4(tm3310)* (b). We did not detect a significant difference between wild-type and *ttll-4*. Bars = 10 µm.

Supplementary figure S2. Ciliary morphology of *ttll-4*. ASH and ASI were visualised by expressing a *sra-6*::GFP transgene in wild-type (a) and *ttll-4(tm3310)* (b). We did not detect a significant difference between wild-type and *ttll-4* mutants. Bars = 5 µm.

Supplementary figure S3. TEM images of amphid channel cilia. Shown are low (left) and high (right) magnification TEM images of the amphid sensory pore from serial cross sections. Images are representative of four pores analysed for each strain. Wild type and *ttll-4* mutant amphid channels possess ten ciliary axonemes, each with a DS, MS, transition zone (TZ), and periciliary membrane compartment (PCMC). Illustration shows the amphid channel in cross section and longitudinal orientations. Note that only three of the ten axonemes are shown for simplicity in the longitudinal illustration. Boxed numbers indicate the position of the section relative to the most anterior section (at '0'); section positions are also indicated in the illustration. Scale bars = 200 nm (large panels) and 100 nm (small panels).

Supplementary figure S4. Tubulin glutamylation is modified by p38/MAPK signalling pathway. (a-d) Tubulin glutamylation of the mutant of p38 MAPK and SAPK/JNK MAPK pathway. (a) Wild-type, (b) *nsy-1(ag3)*, (c) *sek-1(km4)*, (d) *pmk-1(km25)*. (e) Quantified summary of glutamylation signals. Relative intensity to N2 (Y-axis) under various genetic background. N2 (wild type), *ttll-4(tm3310)*—no signal, *ttll-4(tm3310)*; *tzIs1580*[*che-2*::TTLL-4 *rol-6(+)*]—rescued strain. Mutants of p38 MAPK

pathway (*tax-4(p678)*, *unc-43(e408lf)*, *nsy-1(ag3)*, *sek-1(km4)*, and *pmk-1(km25)*) showed a significant increase to wild-type. On the other hand, those of SAPK/JNK MAPK pathway (*mlk-1(ok2471)*, *mek-1(ks54)*, *kgb-1(um3) kgb-2(km16)* and *jnk-1(gk7)*) did not show any differences. Bars = 5 μm.

Supplementary figure S5. Double staining of activated p38 and GT335. (a-c) Results of immunohistochemical analysis of the *C. elegans* head tip using anti-activated p38 (a and d), GT335 (b and e) and merged images (c and f). Signal intensities were compared between control (a-c) and worms exposed to high osmolality (d-f). Bars = 5 μm.

Supplementary figure S6. Amino acid comparison of *C. elegans* and human TTLL family members. Putative MAPK phosphorylation sites (Ser/Thr)-Pro are indicated in bold lettering. The position of Thr^{446}, which is essential for the starvation-induced activation, is indicated by an arrow. *C. elegans* TTLL-4 and human TTLL4 have conserved (Ser/Thr)-Pro at this position (blue/red). Among other members, only human TTLL8 (tubulin glycine-ligase) has a conserved Thr-Pro at this position (green). TTLL-4, TTLL-5, TTLL-9, TTLL-11, TTLL-12, and TTLL-15 are *C. elegans* proteins. hTTLL4, hTTLL1, hTTLL9, hTTLL5, hTTLL2, hTTLL13, hTTLL7, hTTLL11, hTTLL3, hTTLL8, hTTLL12, hTTLL10, and hTTL are human proteins. The orthologues of *C. elegans* and human are aligned up and down (indicated by the lines).

Supplementary figure S7. IFT velocity analysis in the phasmid channel cilia of *ttll-4* mutants. (a) Representative images and kymographs of OSM-6::GFP in wild-type (left) and *ttll-4* (right) worms. Schematics show MS (1) and DS (2) regions from which MS and DS kymographs and IFT rates were determined. Numbers indicate length of cilia as determined by the OSM-6::GFP marker. (b) Histogram of transport velocities in the middle segment. The number of particles moving at given velocity ranges is shown. (c)

Table of summarised result. The *ttll-4* mutant had decreased IFT particle velocity in both MS and DS. N = number of particles measured. P values were derived from a Student's t-test after comparing with the wild-type control.

Supplemental figure S8. Histogram of transport velocities. Left: histogram of transport velocities. The number of particles moving at the indicated ranges of velocity are shown. (a) *pmk-1(km25)*, (b) *ttll-4(tm3310)*; [*che-2*::TTLL-4(Wild-type)], (c) *ttll-4(tm3310)*; [*che-2*::TTLL-4(T446A)]. Blue bars indicate the velocities of well-fed worms. Red bars indicate those of starved worms. Right: fluorescence micrographs with corresponding schematic showing lines used to generate kymographs along the MS.

Supplemental figure S9. The *ttll-4* mutant shows behavioural abnormalities. (a) Nose touch assay, (b) chemotaxis assay for NaCl, (c) osmotic avoidance assay. The *ttll-4* mutants are less sensitive to all three sensory behaviour assays. Single asterisk (*$p < 0.05$) and double asterisk (**$p < 0.01$) indicates the difference from wild-type N2.

Supplementary References

1. Hart, A. C. Behavior. *WormBook*, 1-67 (2006).
2. Saeki, S., Yamamoto, M. & Iino, Y. Plasticity of chemotaxis revealed by paired presentation of a chemoattractant and starvation in the nematode *Caenorhabditis elegans. J Exp Biol* **204**, 1757-1764 (2001).
3. Fujiwara, M., Ishihara, T. & Katsura, I. A novel WD40 protein, CHE-2, acts cell-autonomously in the formation of *C. elegans* sensory cilia. *Development* **126**, 4839-4848 (1999).
4. Starich, T. A. *et al.* Mutations affecting the chemosensory neurons of *Caenorhabditis elegans. Genetics* **139**, 171-188 (1995).
5. Cevik, S. *et al.* Joubert syndrome Arl13b functions at ciliary membranes and stabilizes protein transport in *Caenorhabditis elegans. J Cell Biol* **188**, 953-969, doi:10.1083/jcb.200908133 (2010).

Supplementary Figure S1

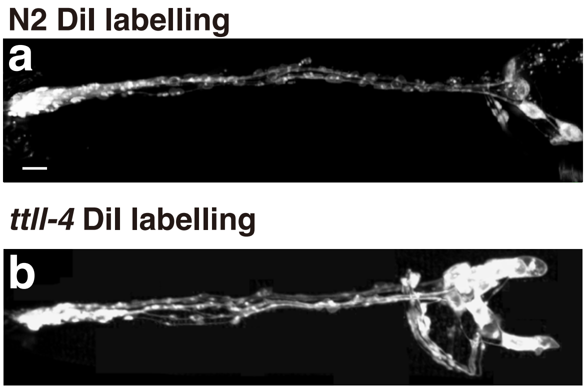

Supplementary Figure S2

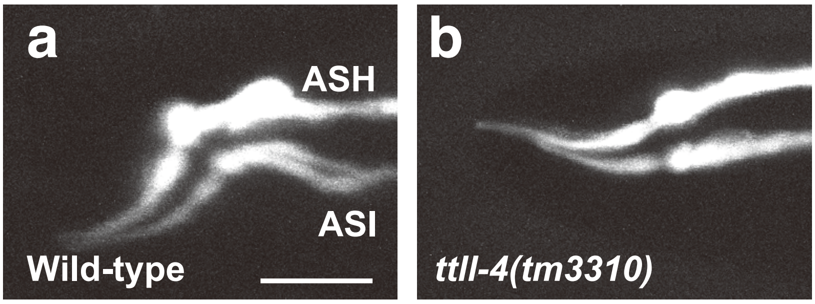

Supplementary Figure S3

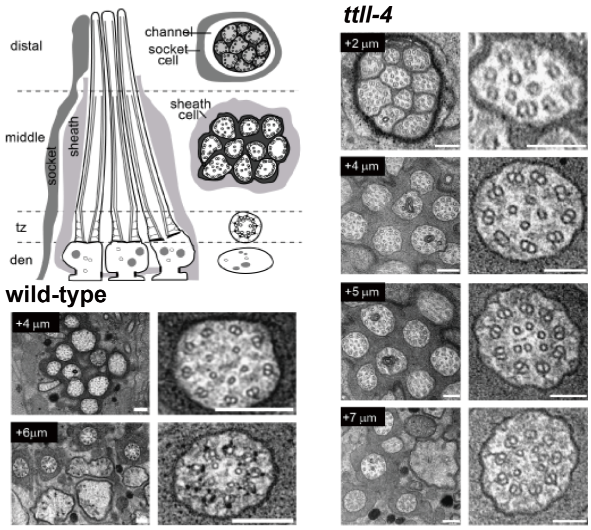

Supplementary Figure S4

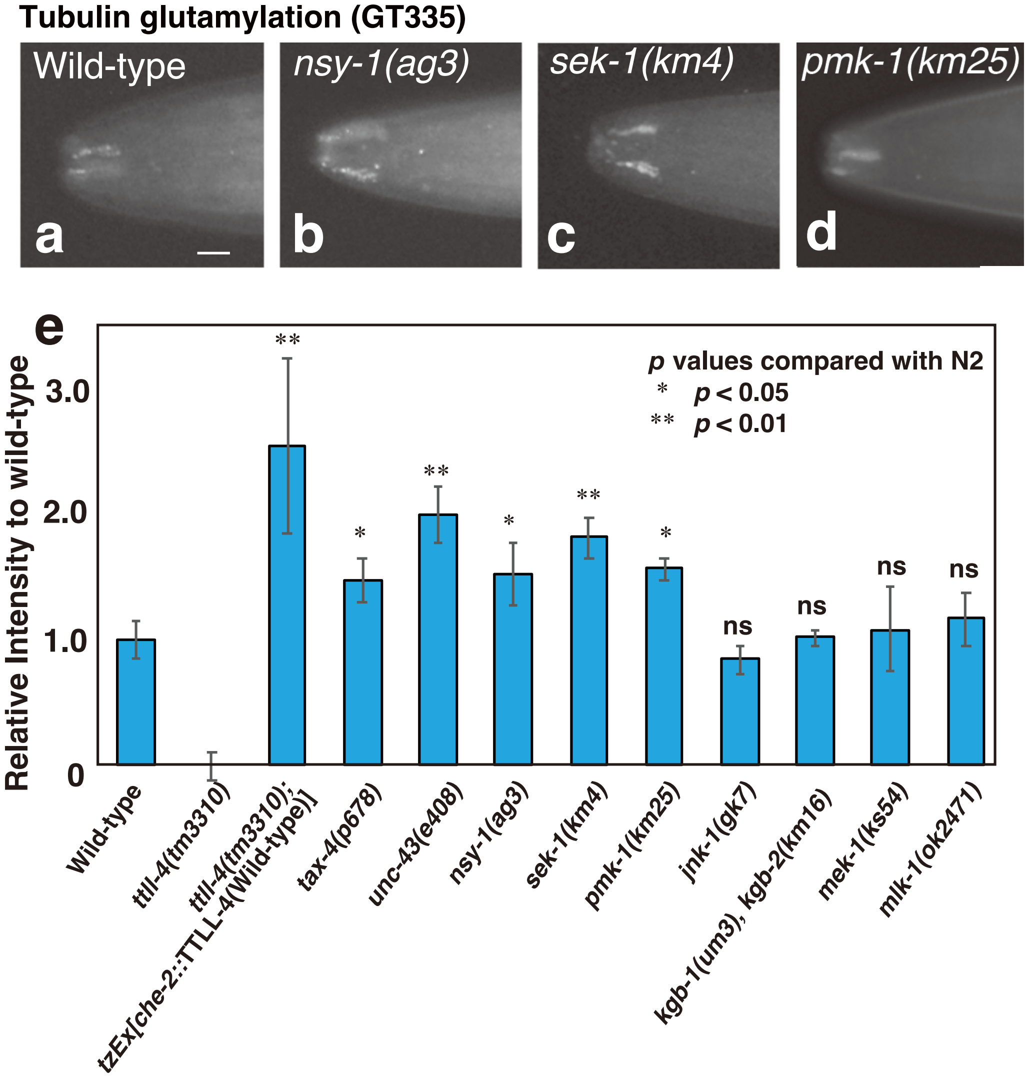

Supplementary Figure S5

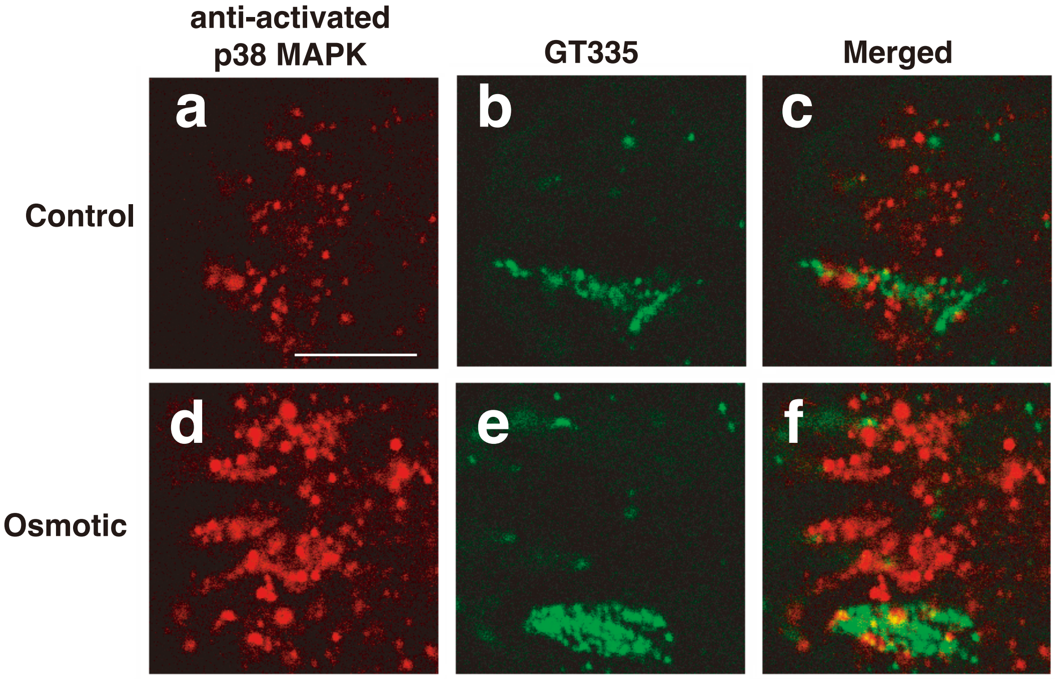

Supplementary Figure S6

```
                                                                   T446
                                                                    ↓
                         420       430       440                       450       460       470
TTLL-4           FICYELFGIDIILDEDYKPWLLEVNISPSLHSG------------------TPLDVSVKAPLAKDVLNLAGVYVPPSFDKLS-----
hTTLL4           YSCHELFGFDIMLDENLKPWVLEVNISPSLHSS------------------SPLDISIKGQMIRDLLNLAGFVLPNAEDII------
TTLL-15           PQYFELSRFDFVVDDQLNVFLMEANMSPNLSSG-----------------HFKQNQILYEQVLMNIFSLTGISTPITKEADI-----
hTTLL1            HCFECYGYDIIIDDKLKPWLIEVNASPSLTSS------------------TANDRILKYNLINDTLNIAVPNGEIPDC--------
hTTLL9            HCFELYGYDILIDQDLKPWLLEVNASPSLTAS------------------SQEDYELKTCLLEDTLHVVDMEARLTG----------
TTLL-9            HCFELYGYDILFDENLKPWLLEVNASPSLTAS------------------SQEDFELKYRILNHMIDVLDIEKKLI----------
hTTLL5           SSCFELYGFDVLIDSTLKPWLLEVNLSPSLACD------------------APLDLKIKASMISDMFTVVGFVCQDP----------
TTLL-5           CTNFELFGFDVLVDQALKPWLLEVNLSPSLACD------------------APLDSLLKTRLIADLLNLACVPLLDRKII-------
hTTLL2           ANCFELFGFDILIDDNLKPWLLEVNYSPALTLD------------------CSTDVLVKRKLVHDIIDLIYLNGLRNEGR-------
hTTLL6           SACFEILGFDILLDHKLKPWLLEVNHSPSFSTD------------------SRLDKEVKDGLLYDTLVLINLESCDK----------
hTTLL13           ACFEILGFDILLDHKLKPWLLEVNHSPSFTTD------------------SCLDQEVKDALLCDAMTLVNLRGCDKRK--------
hTTLL7            VCFEVLGFDILLDRKLKPWLLEINRAPSFGTD------------------QKIDYDVKRGVLLNALKLLNIRTSDKRRN-------
hTTLL11           TCFQILGFDILLMKNLKPILLEVNANPSMRIEHEHELSPGVFEN----VPSLVDEEVKVAVIRDTLRLMDPLKKKRENQ-------
TTLL-11           QCFQIMGFDVMIREDGTPILLEVNAAPSLTADHIVPHPGRTLLEGGQRVRSIVDEVIKIPLVRDTLLLVLGLMEEE----------
hTTLL3            ASFELYGADFVFGEDFQPWLIEINASPTMAPS------------------TAVTARLCAGVQADTLRVVIDRMLDR----------
hTTLL8            NSFELYGADFVLGRDFRPWLIEINSSPTMHPS------------------TPVTAQLCAQVQEDTIKVAVDRSCDI----------
hTTLL12           SSRAMYAVDLMLKWDNGPDGRRVMQPQILEVN------------------FNPDCERACRYHPTFFNDVFSTLGA-----------
TTLL-12           QSRAMYGVDIMLQHGD----NDVIKSTLLEIN------------------FMPDTTRACQYYPDFADTVFETLFLDEIDPTKVTPI
hTTLL10           YFDLIGCDFLIDDNFKVWLLEMNSNPALHTN------------------CEVLKEVIPGVVIETLDLVLETFRKSL---------
hTTL              YQSFQLFGFDFMVDEELKVWLIEVNGAPACAQK--------------------LYAELCQGIVDIAISSVFPPPDVEQP--------
```

Supplementary Figure S7

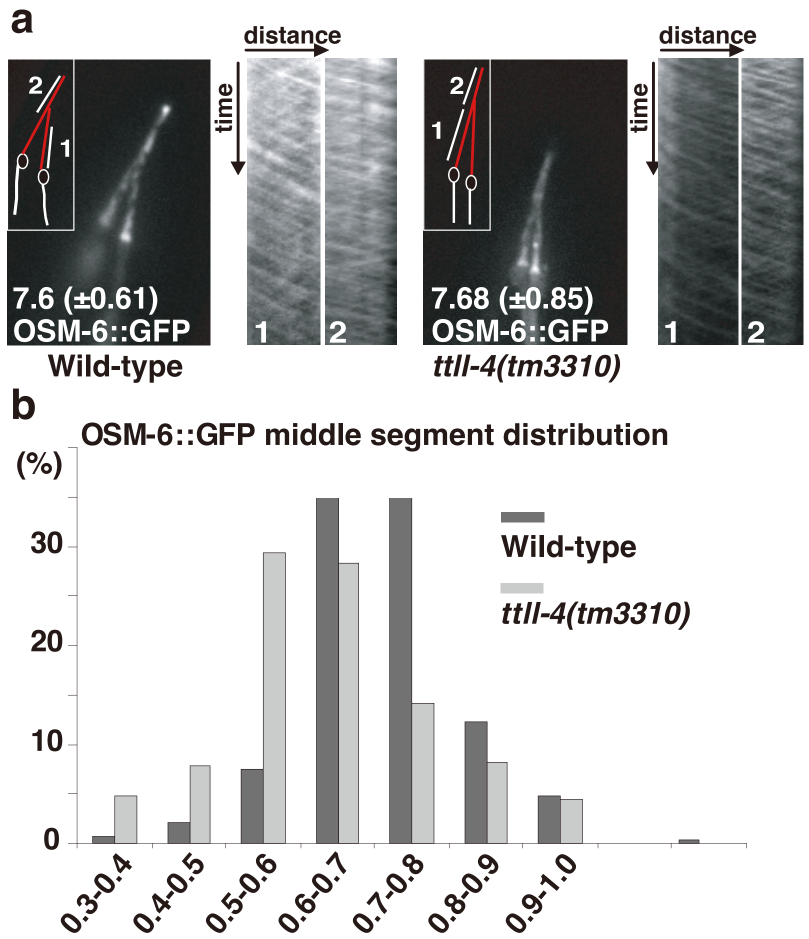

c

		Middle segment		*t*-test
IFT proteins	Strains	Average	n/N	
OSM-6::GFP	Wild-type	0.91±0.10	146/8	
	ttll-4 (tm3310)	0.63±0.13	268/7	< 0.001

		Distal segment		*t*-test
IFT proteins	Strains	Average	n/N	
OSM-6::GFP	Wild-type	1.32±0.12	35/4	
	ttll-4 (tm3310)	1.13±0.25	45/14	< 0.001

Supplementary Figure S8

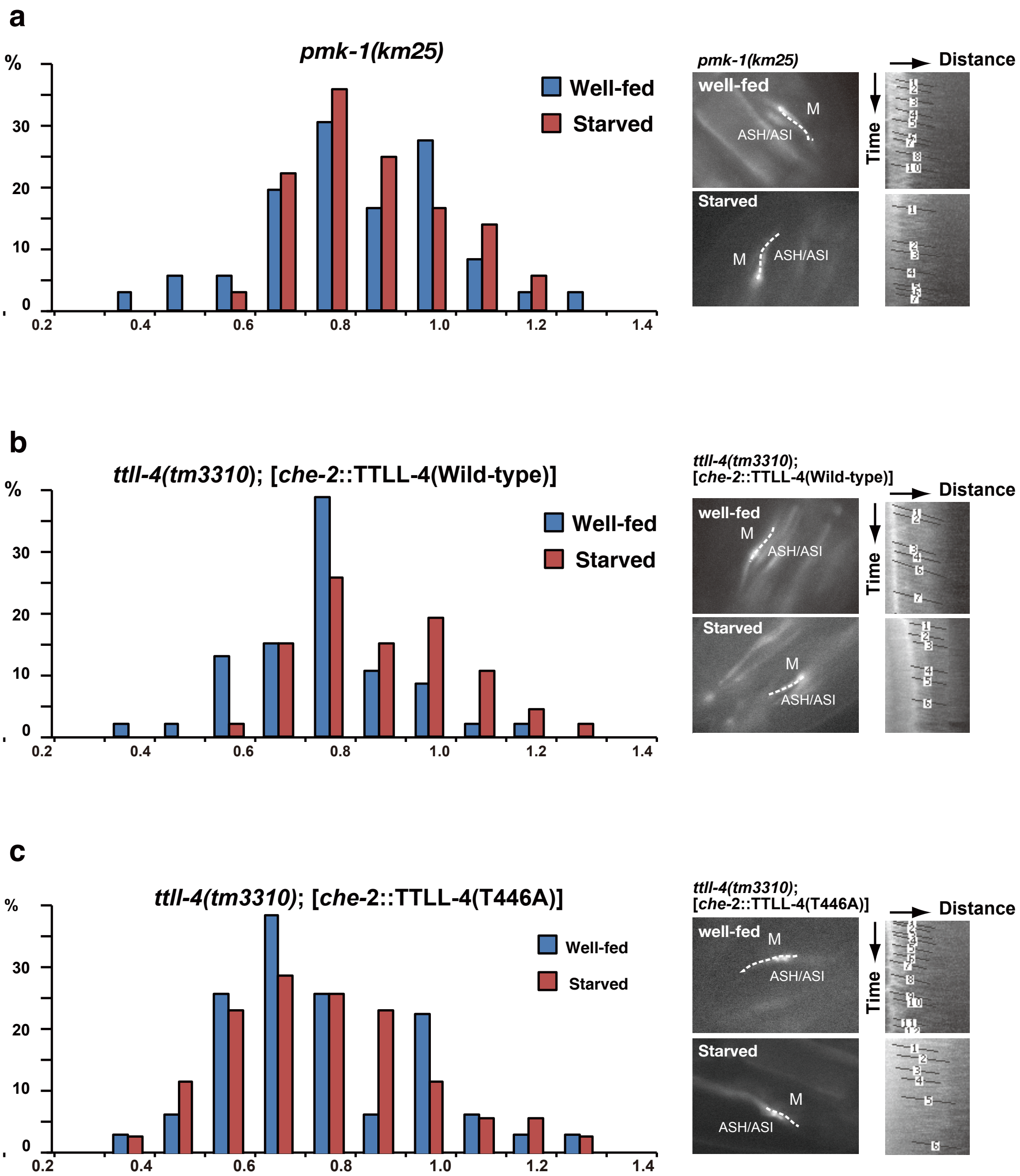

Supplementary Figure S9

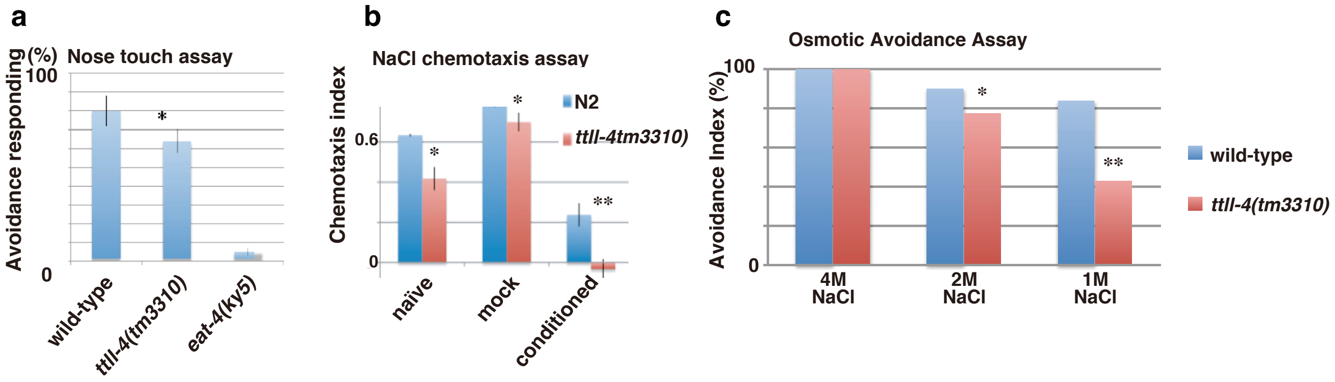

Supplement: Supplementary file 1 — Supplementary information [file 41598_2018_26694_MOESM1_ESM.pdf]
